# RBBP7, regulated by SP1, enhances the Warburg effect to facilitate the proliferation of hepatocellular carcinoma cells via PI3K/AKT signaling

**DOI:** 10.1186/s12967-024-04964-8

**Published:** 2024-02-18

**Authors:** Yuan Fang, WeiQiang Tang, Siming Qu, ZhiTao Li, XiaoLi Zhang, YingLei Miao, Zhong Zeng, HanFei Huang

**Affiliations:** 1 Organ Transplantation Center, The First Affiliated Hospital of Kunming Medical University, 295 Xichang Road, Kunming, 650032 Yunnan China; 2 Institute of Clinical Medicine, Hengyang Medical School, The First Affiliated Hospital, University of South China, Hengyang, Hunan People’s Republic of China; 3 Gastrointestinal and Hernia Surgery, The First Affiliated Hospital of Kunming Medical University, Kunming, Yunnan People’s Republic of China; 4 Department of Gastroenterology, The First Affiliated Hospital of Kunming Medical University, Yunnan, People’s Republic of China; 5 Yunnan Province Clinical Research Center for Digestive Diseases, Yunnan, People’s Republic of China

**Keywords:** RBBP7, Glycolysis, SP1, Hepatocellular carcinoma, Proliferation

## Abstract

**Background:**

Hepatocellular carcinoma (HCC) is characterized by aggressive progression and elevated mortality rates. This study aimed to investigate the regulatory effects of RBBP7 on HCC pathogenesis and the underlying mechanisms.

**Methods:**

The expression and clinical feature of RBBP7 were evaluated using bioinformatics analysis and the assessment of clinical HCC samples. CCK8 and colony formation were employed to estimate cell proliferation function of RBBP7. Aerobic glycolysis levels of RBBP7 were evaluated by measuring ATP levels, lactic acid production, glucose uptake capacity, and the expression of relevant enzymes (PFKM, PKM2, and LDHA). The phosphorylation levels in PI3K/AKT signaling were measured by western blotting. The regulatory effect of transcription factors of specificity protein 1 (SP1) on *RBBP7* mRNA expression was confirmed in dual-luciferase reporter assays and chromatin immunoprecipitation experiments. The proliferation- and glycolysis-associated proteins were assessed using immunofluorescence staining in vivo*.*

**Results:**

We found that RBBP7 is expressed at high levels in HCC and predicts poor survival. Functional assays showed that RBBP7 promoted HCC proliferation and glycolysis. Mechanistically, it was demonstrated that RBBP7 activates the PI3K/AKT pathway, a crucial pathway in glycolysis, contributing to the progression of HCC. The outcomes of the dual-luciferase assay further confirmed that SP1 is capable of activating the promoter of RBBP7.

**Conclusions:**

RBBP7, which is up-regulated by SP1, promotes HCC cell proliferation and glycolysis through the PI3K/AKT pathway. The findings of this study suggest that RBBP7 is a potential biomarker for HCC.

**Supplementary Information:**

The online version contains supplementary material available at 10.1186/s12967-024-04964-8.

## Background

Hepatocellular carcinoma (HCC), which is the predominant form of primary liver cancer, is associated with high cancer-related mortality rates [1]. Surgery is the most efficacious treatment for patients with HCC. However, approximately 80% of patients with HCC are not eligible for surgery as they are diagnosed with advanced-stage tumors [2]. Previous studies have aimed to develop non-invasive biomarkers to detect early cancer and/or determine cancer risk and consequently decrease HCC-related mortality rates [3, 4]. The pathological mechanisms underlying HCC progression have not been elucidated. Hence, there is an urgent need to identify and develop effective diagnostic biomarkers and therapeutic targets for HCC.

The activation of aerobic glycolysis is a distinctive characteristic of HCC. Aerobic glycolysis modulates liver cancer cell proliferation, invasion, and drug resistance [5]. Although the efficiency of adenosine triphosphate (ATP) production during aerobic glycolysis is low, the supply of ATP remains as high as 50–70% in different tumors [6]. Glycolysis involves various enzymes, including PFKM, PKM, and LDHA, which are reported to be potential therapeutic targets [7]. Aerobic glycolysis has a major role in HCC pathogenesis and can be regulated by various pathways, including the AMPK, PIK3CA/AKT1, and non-coding RNA pathways [8].

Recent studies have reported the mechanism underlying aerobic glycolysis activation in HCC. The dysregulation of glycolysis-related gene expression can activate aerobic glycolysis in HCC [9]. Here, we focused on retinoblastoma-binding protein 7 (RBBP7), which is a highly conserved in WD-repeat subfamily that are primarily expressed in the nucleus [10]. Previous studies have reported the role of RBBP7 in tumor initiation and progression although its role in HCC pathogenesis is unclear [11, 12]. Chen et al*.* reported that RBBP7 expression in liver tumor tissues is significantly upregulated when compared with that in non-tumorous liver tissues [13]. Nevertheless, the mechanism by which RBBP7 regulates HCC phenotype still largely unknown.

Based on the abovementioned findings, in HCC, RBBP7 may be used as a target for HCC development. Through this research, we aim to explore the impact of RBBP7 expression in HCC. Our findings offer novel insights that could contribute to the advancement of therapeutic approaches for HCC.

## Methods

### Cell culture

Hep3B, Huh7, PLCPRF5, Li7, and THLE2 cells were purchased from ProCell (Wuhan, China). The THLE2, PLCPRF5 and Hep3B were cultured with Minimum Essential Medium. Huh7 was cultured with Dulbecco’s Modified Eagle Medium. Li7 was cultured with Roswell Park Memorial Institute-1640. These medium was added with 10% FBS and 1% penicillin–streptomycin. Incubation was carried out in an environment with 5% CO_2_ at a temperature of 37 °C.

### Lentiviral infection and transient transfection

To overexpress RBBP7, 1410 bp of RBBP7 (NM_001198719.2) was cloned into the lentiviral transfer plasmid pLVX-Puro to generate recombinant pLVX-Puro-RBBP7. The pLVX-Puro-RBBP7 plasmid (1000 ng) was co-transfected with psPAX2 (100 ng) and pMD2.G (900 ng) to generate the indicated lentivirus. For RBBP7 RNAi, shRNA plasmids (shRBBP7-1, -2, and-3) were obtained from GenePharma (Shanghai, China) (shRBBP7s used are exhibited in Additional file 1: Table S1). Lipofectamine 2000 (Invitrogen, CA, USA) was performed for transient-transfection in accordance with the protocol. Overexpression of specificity protein 1 (SP1) (NM_138473.3) was cloned into pCDNA3.1( +).

### Bioinformatics assay

GEPIA2 (http://gepia2.cancer-pku.cn/#index) is a dataset web which uses standardized analyses of RNA-seq data. We used the database to study RBBP7 expression, survival, correlation, and clinical information analysis. GEO database (GSE135631) was performed to explore RBBP7 expression. Ggplot2 of R studio (4.2.1) was used to analyze clinical information on HCC retrieved from TCGA. To perform receiver operating characteristic (ROC) analysis, the expression data were obtained from TCGA database (https://portal.gdc.cancer.gov) and TCGA-liver hepatocellular carcinoma (LIHC) project. The RNA-seq data were aligned using the STAR aligner, and the TPM format of the data was extracted. The data were subjected to ROC analysis using the pROC package, and the results were visualized using ggplot2.

### Real-time qPCR (RT-qPCR)

Cells were subjected to TRIzol reagent (Thermo Fisher Scientific) for RNA extraction, followed by cDNA synthesis using the PrimeScript RT Reagent Kit (TaKaRa, Kusatsu, Japan) as per the protocol.

RT-qPCR was conducted using the following specific primers: RBBP7-Forward 5ʹ-CACGTGCATTTGTCTTCCCG-3ʹ, RBBP7-Reverse 5ʹ-CCCCAGCACTAGCCAATGAA-3ʹ; SP1-Forward 5ʹ- GAGGAGGAGGGCAGGAGTC-3ʹ, SP1-Reverse 5ʹ- AGTTGTGTGGCTGTGAGGTC-3ʹ; β-actin-Forward 5ʹ- CCTTCCTTCCTGGGCATGG-3ʹ, β-actin-Reverse 5ʹ- GATCTTCATTGTGCTGGGTGC -3ʹ. RT-qPCR was conducted using FastStart Universal SYBR Green Master (Roche Diagnostics, Mannheim, Germany) on a RochLightCycle 480II PCR. The expression levels of relative mRNA of genes were determined by the 2^(− ΔΔCt)^ method.

### Western blotting

Total protein was harvested from cells lysed using RIPA buffer (Solarbio, Beijing, China) supplemented with a protease and phosphatase inhibitor complex (Solarbio). Protein concentrations were determined using the BCA Protein Assay Kit (Beyotime, Shanghai, China). The proteins were subsequently separated on 10% SDS-PAGE gels. During the transfer of the proteins to the PVDF membranes, a 10% non-fat milk solution was performed to block the membranes at room temperature (2 h). The membranes were analyzed using an AI600 Imager (GE Healthcare Life Sciences, USA). Densitometric analyses were performed using ImageJ. The antibodies and their dilutions are listed in Additional file 1: Table S2.

### Cell proliferation

To assess cell proliferation, the kit of CCK-8 (C0039, Beyotime) was implemented according to the protocol. At the indicated time points (0, 12, 24, 48, and 72 h) for CCK-8 examination, the optical density (OD) of the samples at 450 nm was measured using a microplate reader (DNM-9602, Perlong, USA). For colony formation, cells were inoculated in 3.5 cm dishes at 800 cells per well for 2 weeks and subjected to crystal violet staining (0.1%). Subsequently, the colonies were imaged and counted.

### ATP, lactate, and glucose uptake assay

ATP synthesis, lactate levels, and glucose uptake were measured using the ATP Colorimetric Assay kit, Lactate Assay Kit II, and Glucose Uptake Colorimetric Assay kit, respectively, following the manufacturer’s instructions (Beyotime). NAD^+^/NADH Colorimetric Assay Kit (WST-8) was following the manufacturer’s instructions (Elabscience).

### Dual-luciferase reporter assays

The 600 bp sequence of RBBP7 was cloned to construct the WT-RBBP7-PGL3-Basic plasmid. Relative luciferase activity was measured using the Dual-Luciferase Assay Kit (Beyotime) after 48 h. MUT- RBBP7-PGL3- Basic was constructed as a control. After 48 h, the relative luciferase activity was measured by Dual-Luciferase Assay Kit (E1910, Promega, Beijing, China).

### Chromatin immunoprecipitation (ChIP) assay

The ChIP assay was conducted the SimpleChIP^®^ Plus Sonication Chromatin IP Kit (56383; CST) following to the protocol. In brief, 293T and Hep3B cells were exposed to 1% formaldehyde for around 10 min. The reaction was terminated by treating glycine to the cells for 5 min. Chromatin fragments were generated in cell lysates upon sonication, which immunoprecipitation was carried out using anti-RBBP7 antibody or control rabbit IgG with the treated cells. The reverse-crosslinked complexes were purified, and the amplified DNA was quantified using RT-qPCR. The fold enrichment of the bound region was determined as the quantity of DNA relative to the total DNA.

### Animal experiments

Twenty-four 4–6-week-old BALB/c nude mice (Shanghai SLAC Laboratory Animal Co. Ltd.) were fed at the animal medical laboratory of Kunming Medical University and used to construct tumor models in vivo. For subcutaneous cell line xenografts, 3 × 10^7^ Hep3B cells were suspended in 100 μL of PBS and then injected subcutaneously into mice.

### Immunofluorescence staining

Paraffin sections were boiled in EDTA for 10 min after dewaxing and rehydration. Subsequently, they were treated with the primary antibody overnight at 4 °C. The recommended secondary antibodies were selected for treatment at room temperature.

### Statistical analysis

Statistical analyses were conducted using SPSS (version 26.0), and a Student’s t-test was performed to evaluate the differences between groups. A significance level of *P* < 0.05 was considered statistically significant.

## Results

### RBBP7 is high expression and mean poor prognosis in HCC

**Fig. 1:**
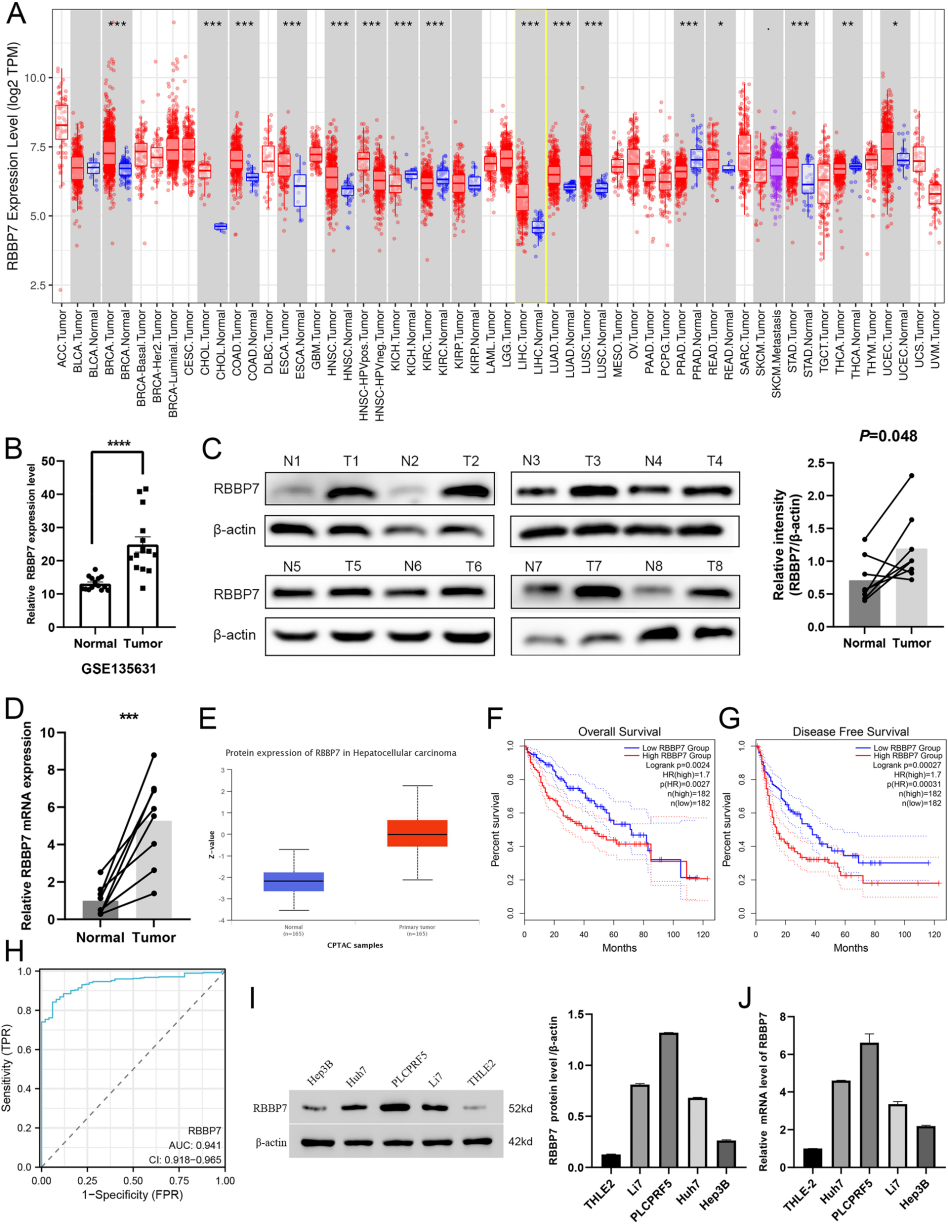
In HCC, RBBP7 is overexpressed and its overexpression is associated with poor prognosis. *, *P* < 0.05, **, *P* < 0.01, ***, *P* < 0.001. **A**. The levels of RBBP7 in various cancer types from TCGA were determined using TIMER. **B**. The mRNA expression levels of RBBP7 in HCC tumor samples and matched non-tumor samples (n = 15) were obtained from the GEO profile (GSE135631). **C**. The protein expression levels of RBBP7 in HCC tumor samples and matched non-tumor samples (n = 8) were obtained from the clinical. **D**. The mRNA expression levels of RBBP7 in HCC tumor samples and matched non-tumor samples (n = 8) were obtained from the clinical. **E**. The protein expression levels of RBBP7 in HCC tumor samples and matched non-tumor samples (n = 165) were obtained from UCLAN. **F**–**G**. Analysis of overall survival (**F**) and disease-free survival (**G**) of patients with HCC from the GEPIA2 website. H. ROC analysis predicting the sensitivity of RBBP7 in HCC based on TCGA data. **I**. The mRNA levels of RBBP7 in THLE-2 and tumor cells (Huh7, PLCPRF5, Li7, and Hep3B cells). **J**. The protein levels of RBBP7 in THLE-2 and tumor cells (Huh7, PLCPRF5, Li7, and Hep3B cells)

The expression of RBBP7 in various tumor types was examined using TIMER (https://cistrome.shinyapps.io/timer/). RBBP7 expression was significantly upregulated in most cancers (Fig. 1A). In particular, the expression of RBBP7 was significantly upregulated in breast cancer, cholangiocarcinoma, colon adenocarcinoma, esophageal carcinoma, head and neck squamous cell carcinoma, LIHC, lung adenocarcinoma, lung squamous cell carcinoma, rectum adenocarcinoma, stomach adenocarcinoma (STAD), and uterine corpus endometrial carcinoma. RBBP7 was downregulated in kidney chromophobe, kidney renal clear cell carcinoma, prostate adenocarcinoma, and STAD. In the GES135631 dataset, the expression of RBBP7 in HCC tissues was upregulated when compared with that in adjacent non-cancerous tissues (*P* < 0.0001) (n = 15) (Fig. 1B). To verified this bioinformation result, we collected 8 clinical tumor samples with matched normal liver tissues for Western blotting and RT-qPCR. In protein and mRNA level, RBBP7 was higher in tumor group than normal. (*P* < 0.05, Fig. 1C, D) Additionally, RBBP7 protein expression was higher expression in CPTAC (https://ualcan.path.uab.edu/index.html) HCC samples (Fig. 1E) [14]. Survival curves from GEPIA2 showed that up-regulated RBBP7 was correlated with poorer OS and DFS. (Fig. 1F, G) The region under the ROC curve for RBBP7 was calculated to be 0.941 (CI 0.918–0.965), which is considerably high. Therefore, RBBP7 holds promise as a potential biomarker for diagnosing HCC. (Fig. 1H) Hence, we focused on evaluating the role of RBBP7 in HCC. Both mRNA and protein of RBBP7 were notably upregulated in cell lines of HCC compared to THLE2. PLCPRF5 had the highest expression levels and Hep3B had the lowest expression levels in HCC cell lines. Based on this finding, we selected these two cell lines for further investigation (Fig. 1I, J).

### In HCC, up-regulated of RBBP7 enhanced growth and glycolysis cells in vitro

**Fig. 2:**
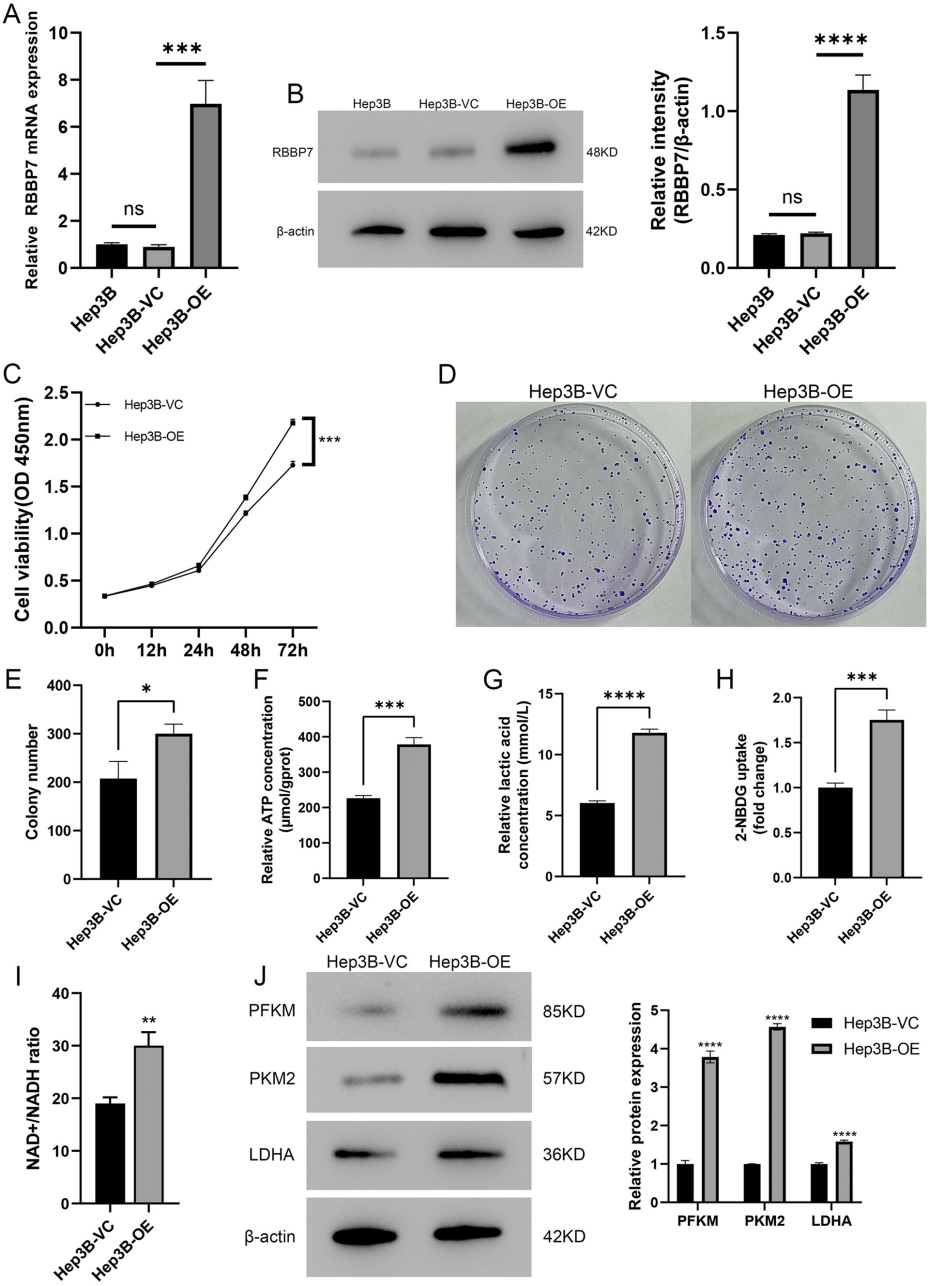
RBBP7 upregulation enhanced HCC proliferation and glycolysis in vitro. Compared with Hep3B-VC group *, *P* < 0.05, **, *P* < 0.01, ***, *P* < 0.001. ****, *P* < 0.0001. **A**, **B**. A stable RBBP7 overexpression model was established in the Hep3B. The upregulated expression of RBBP7 was confirmed through western blotting analysis and RT-qPCR. **C**. The CCK-8 assay was performed to examine cell growth after RBBP7 overexpression. **D**, **E**. A colony formation assay was conducted using Hep3B overexpression cells. **F**. ATP concentration in RBBP7 overexpression and vector control cells. **G**. Lactic acid concentration in upregulation of RBBP7 and vector control cells. **H**. Glucose uptake capacity of upregulation of RBBP7 and vector control cells. **I**. NAD^+^/NADH colorimetric assay of upregulation of RBBP7 and vector control cells. **J**. Glycolysis-associated proteins (PFKM, PKM2, and LDHA) in RBBP7-overexpressing and vector control cells

To investigate the RBBP7 function, we transfected Hep3B cells with RBBP7 or a control sequence carried by a lentivirus. RBBP7 were confirmed the up-regulated level of mRNA and protein by qRT-PCR in Hep3B (*P* < 0.01) and western blotting (*P* < 0.01, Fig. 2A, B). CCK-8 assays confirmed that RBBP7 overexpression (OE) significantly enhanced the proliferative potential of Hep3B. (Fig. 2C) The effects of RBBP7 overexpression on Hep3B proliferation were further demonstrated in the colony formation assay (Fig. 2D, E). To determine the effect of RBBP7 expression on glycolysis, ATP, lactic acid, 2-NBDG glucose uptake assays and NAD^+^/NADH colorimetric assay were performed. Notably, we observed a significant increase in ATP levels, lactic acid production, glucose uptake capacity and NAD^+^/NADH upon upregulation of RBBP7 expression. (*P* < 0.01, Fig. 2F–I) Furthermore, glycolysis-associated proteins (PFKM, PKM2, and LDHA) were drastically upregulated following RBBP7 overexpression (*P* < 0.0001, Fig. 2J).

### Low expression of RBBP7 inhibited growth and glycolysis in HCC in vitro

**Fig. 3:**
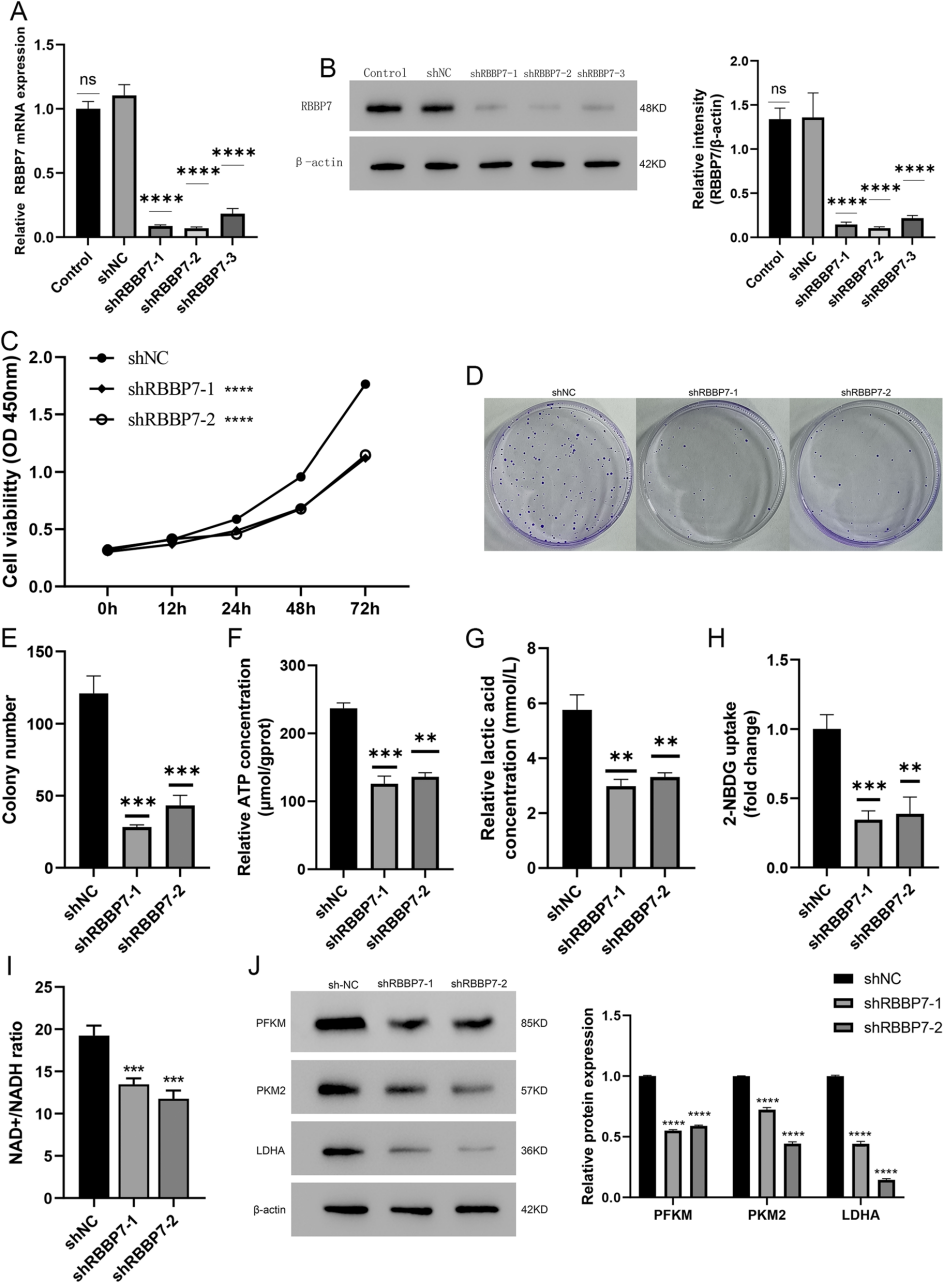
RBBP7 downregulated suppressive HCC cell proliferation and glycolysis in vitro*.* Compared with shNC group *, *P* < 0.05, **, *P* < 0.01, ***, *P* < 0.001. ****, *P* < 0.0001. **A**, **B**. RBBP7 downregulation was established in PLCPRF5 and was successfully verified by western blotting analysis and RT-qPCR. **C**. CCK-8 assay was performed to examine cell growth after RBBP7 knockdown. **D**, **E**. A colony formation assay was conducted using RBBP7 knockdown cells. **F**. ATP concentration in RBBP7 knockdown and vector control. **G**. Lactic acid concentration in RBBP7 knockdown and vector control. **H**. Glucose uptake capacity of RBBP7 knockdown and vector control. **I**. NAD^+^/NADH colorimetric assay of RBBP7 knockdown and vector control. **J**. Expression of glycolysis-associated proteins (PFKM, PKM2, and LDHA) in RBBP7 knockdown and vector control cells

To evaluate the downregulation of RBBP7 in HCC, we stably downregulated the expression of RBBP7 using three shRNAs (shRBBP7-1, shRBBP7-2, and shRBBP7-3) in PLCPRF5. As shown in Fig. 2A, B, shRBBP7-1 and shRBBP7-2 knocked down mRNA and protein expression and were selected for the subsequent experiments (*P* < 0.01). The knockdown of RBBP7 in PLCPRF5 resulted in a significant suppressive of their proliferation potential. (*P* < 0.01, Fig. 3C–E). Meanwhile, we observed that RBBP7 downregulation significantly reduced the ATP levels, lactic acid production, the glucose uptake capacity and the NAD^+^/NADH ratio (*P* < 0.01, Fig. 3F–E).

Glycolysis-associated genes (PFKM, PKM2, and LDHA) were considerably downregulated after RBBP7 knockdown (*P* < 0.0001, Fig. 3I).

**Fig. 4:**
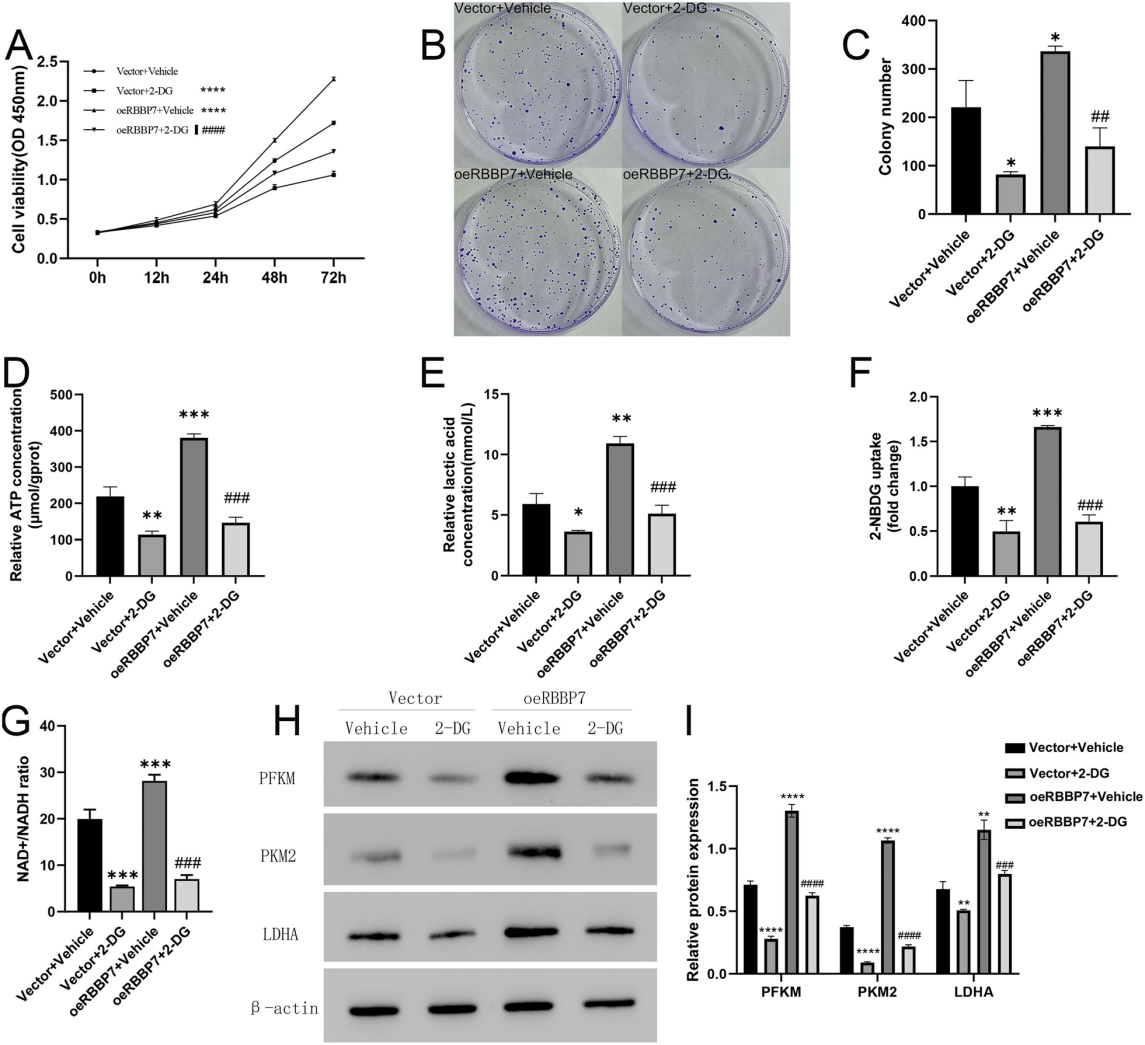
Treatment with 2‐DG could suppressive the effect of RBBP7 facilitate HCC cell proliferation and glycolysis in vitro*.* Compared with shNC group *, *P* < 0.05, **, *P* < 0.01, ***, *P* < 0.001. ****, *P* < 0.0001. Compared with oeRBBP7 + Vehicle group #, *P* < 0.05, ##, *P* < 0.01, ###, *P* < 0.001. ####, *P* < 0.0001. **A**. CCK-8 assay was performed to examine cell growth after RBBP7 overexpression with 2-DG. **B**, **C**. A colony formation assay was conducted using RBBP7 overexpression with 2-DG. **D**. ATP concentration in RBBP7 overexpression with 2-DG. **E**. Lactic acid concentration in RBBP7 overexpression with 2-DG. **F**. Glucose uptake capacity of RBBP7 overexpression with 2-DG. **G**. NAD^+^/NADH colorimetric assay of RBBP7 overexpression with 2-DG. **H**, **I**. Expression of glycolysis-associated proteins (PFKM, PKM2, and LDHA) in RBBP7 overexpression with 2-DG

The results of the CCK8 and colony formation assays revealed that treatment with 1 mM of 2-deoxy-d-glucose (2-DG), a specific inhibitor of glycolysis, for 24 h partially mitigated the glycolysis-induced proliferation of Hep3B cells. Additionally, treatment with 2-DG downregulated the levels of ATP, lactic acid, 2-NBDG uptake, NAD + /NADH ratio, and glycolysis-associated proteins. Meanwhile, RBBP7 OE reversed the inhibitory effects of 2-DG on Hep3B cell proliferation and glycolysis. (*P* < 0.0001, Fig. 4A–I).

### RBBP7 activated the PI3K/AKT pathway in HCC

**Fig. 5:**
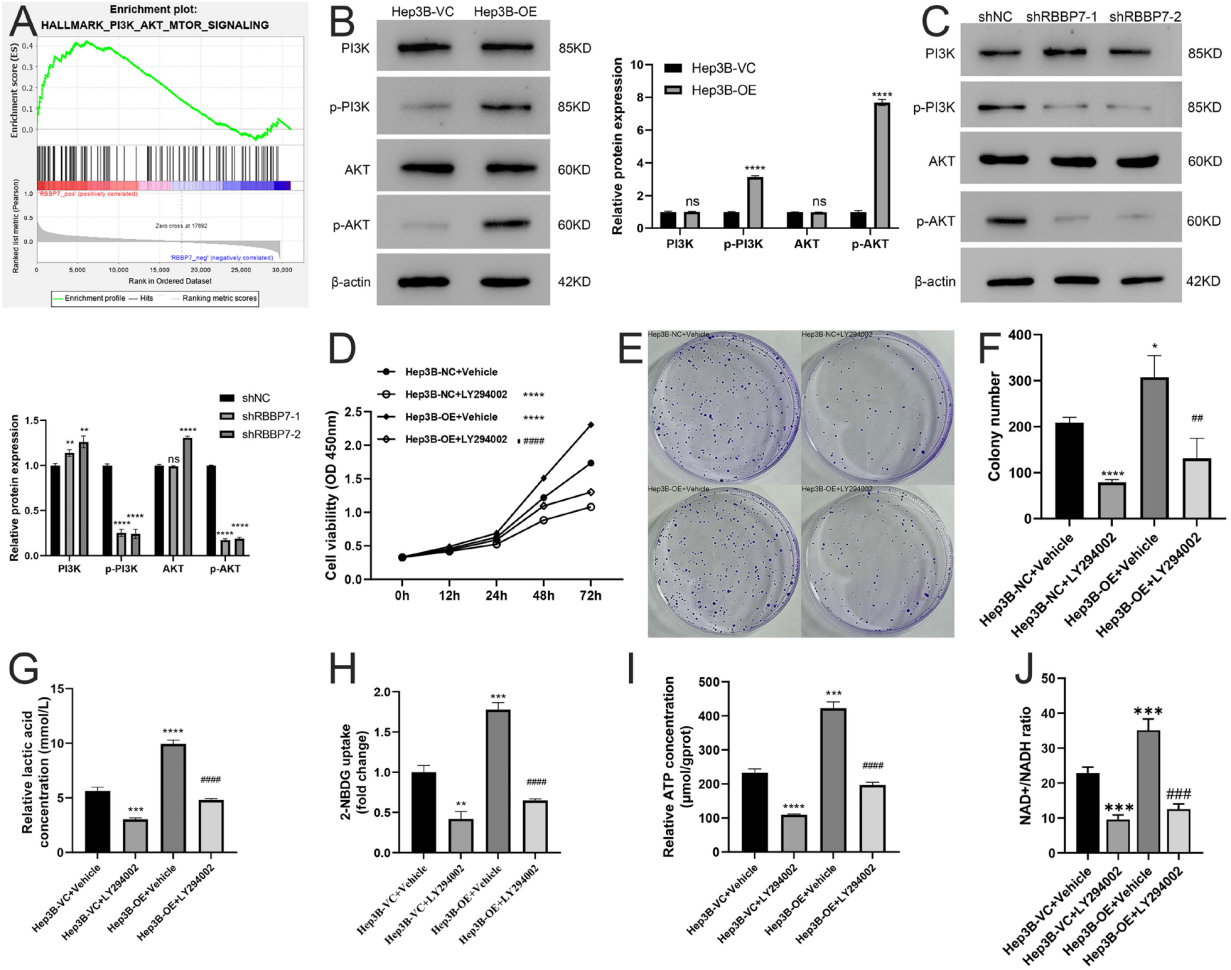
RBBP7 promotes HCC through the PI3K/AKT signaling. Compared with Hep3B-NC + Vehicle group *, *P* < 0.05, **, *P* < 0.01, ***, *P* < 0.001. ****, *P* < 0.0001. Compared with Hep3B-OE + Vehicle group #, *P* < 0.05, ##, *P* < 0.01, ###, *P* < 0.001. ####, *P* < 0.0001. **A**. GSEA was performed to identify the RBBP7 signaling pathway. **B**. PI3K, p-PI3K, AKT, and p-AKT expression in RBBP7 overexpression and vector control cells measured by western blotting. **C**. PI3K, p-PI3K, AKT, and p-AKT expression in RBBP7 knockdown and vector control cells measured by western blotting. **D**. CCK-8 assay was employed to examine the growth of RBBP7-overexpressing cells in the PI3K signaling pathway rescue experiment. **E**, **F**. In the PI3K signaling pathway rescue experiment, a colony formation assay was used to assess the growth of RBBP7-overexpressing cells. **G**. Lactic acid concentration in RBBP7-overexpressing cells during the PI3K pathway rescue experiment. **H**. Glucose uptake capacity in RBBP7-overexpressing cells during PI3K pathway rescue experiment. **I**. ATP concentration in RBBP7-overexpressing cells during the PI3K pathway rescue experiment. **J**. NAD^+^/NADH colorimetric assay in RBBP7-overexpressing cells during PI3K pathway rescue experiment

GSEA was employed to explore molecular mechanisms of RBBP7. PI3K/AKT pathway was correlation with the mechanism of action of RBBP7 (Fig. 5A). The western blotting experiments revealed that the levels of p-PI3K and p-AKT in Hep3B were notably increased in RBBP7 overexpression group. RBBP7 downregulation decreased p-PI3K (*P* < 0.0001; Fig. 5B) and p-AKT expression (*P* < 0.0001, Fig. 5C). These data indicate that RBBP7 functions through the PI3K/AKT signaling. LY294002, a PI3K inhibitor, was performed to investigate the PI3K/AKT signaling pathway for further validation. The influence of RBBP7 overexpression on Hep3B cell proliferation was reversed via LY294002. (*P* < 0.0001, Fig. 5D–F) Likewise, when Hep3B cells were treated with LY294002, the ATP and lactic acid levels and glucose uptake capacity were inhibited which the promotion from RBBP7. (*P* < 0.0001, Fig. 5G–J).

### RBBP7 promotes glycolysis and tumor growth via SP1 in HCC

**Fig. 6:**
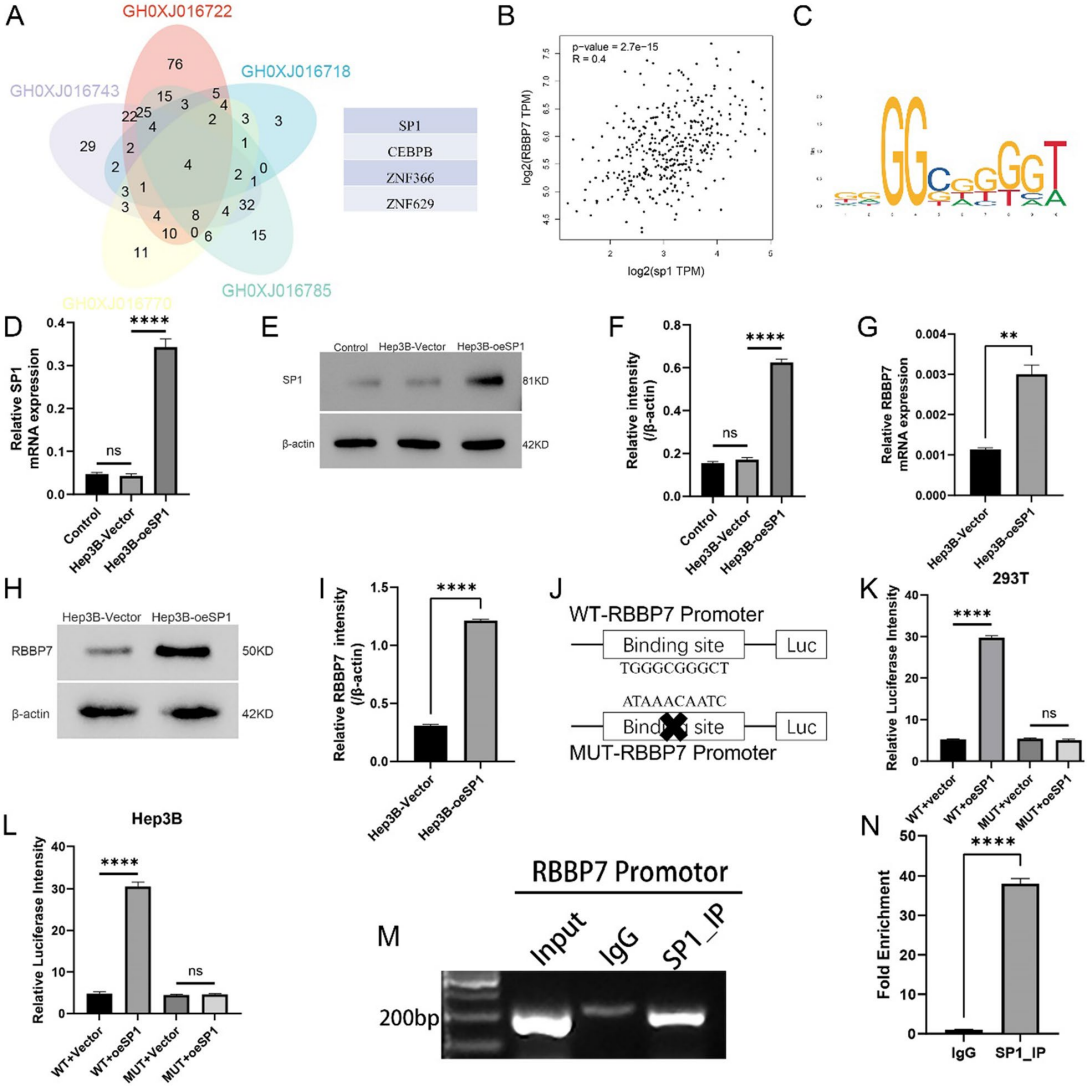
RBBP7 expression is regulated by SP1. *, *P* < 0.05; **, *P* < 0.01; ***, *P* < 0.001; ****, *P* < 0.0001. **A**. GeneCards is used to explore RBBP7-related transcription factors. **B**. Correlation analysis between RBBP7 and SP1 expression. **C**. The JASPAR database was used to predict the putative score for SP1 binding to RBBP7. **D**–**F**. SP1 upregulation was established in the Hep3B and was successfully verified by western blotting analysis and RT-qPCR. **G**–**I**. The RBBP7 level was determined by western blotting in SP1 overexpressing and control group. **J**–**L**. Dual luciferase activity assays were conducted to measure the fluorescence intensity of vector and oeSP1 cells, both with and without mutations in the RBBP7 promoter region, in both 293T and Hep3B cells. **M**, **N**. A ChIP-qPCR assay was used to study the enrichment of RBBP7 binding in SP1

To further search the mechanism of action of RBBP7 in HCC, we employed the GeneHancer Identifier of GeneCards database (https://www.genecards.org/) to identify related transcription factors. Using the VENN diagram, we identified four transcription factors (SP1, CEBPB, ZNF366, and ZNF629) (Fig. 6A). Next, we performed mRNA correlation analysis using GEPIA2 to identify the most relevant transcription factor for RBBP7. (R = 0.4, *P* < 0.0001, Fig. 6B). CEBPB (R = − 0.084, P = 0.11) and ZNF366 (R = − 0.02, P = 0.7) were not correlated with RBBP7. The R value for the correlation between RBBP7 and ZNF629 was 0.42. However, the role of ZNF629 as a transcription factor has not been previously reported (Additional file 1: Fig. S1). Therefore, SP1 was considered to be the potential transcription factor for RBBP7. JASPAR (https://jaspar.genereg.net/) was employed for explore the binding-site of SP1 to the promoter region of RBBP7 (Fig. 6C) [15]. To validate the results of the bioinformatics analysis, we transfected an SP1 overexpression plasmid into Hep3B (P < 0.001, Fig. 6D–F). RBBP7 protein and mRNA expression was upregulated in the SP1 overexpression group (oeSP1) compared to the empty vector (Hep3B-Vector) (*P* < 0.01, Fig. 6G–I). These results imply that SP1 expression is correlated with RBBP7 expression. In order to delve deeper into the functional significance of SP1 binding to the RBBP7 promoter, we inserted a 600bp segment of RBBP7 promoter into the PGL3-luciferase reporter vector and examined the luciferase reporter activity. For control, we predicted and modified the sites with a high probability of RBBP7 binding using consensus motifs from JASPAR.

The overexpression of SP1 in 293T and Hep3B cells significantly increased the activity of the luciferase reporter containing the wild-type (WT) RBBP7 promoter sequences. Nevertheless, the luciferase reporter with mutant-type (MUT) RBBP7 promoter constructs did not show a increased in luciferase activity in response to SP1 overexpression. The ChIP assay results provided further confirmation of the binding of SP1 to the RBBP7 promoter. (Fig. 6M, N).

### SP1 enhanced the function of *RBBP7* in HCC

**Fig. 7:**
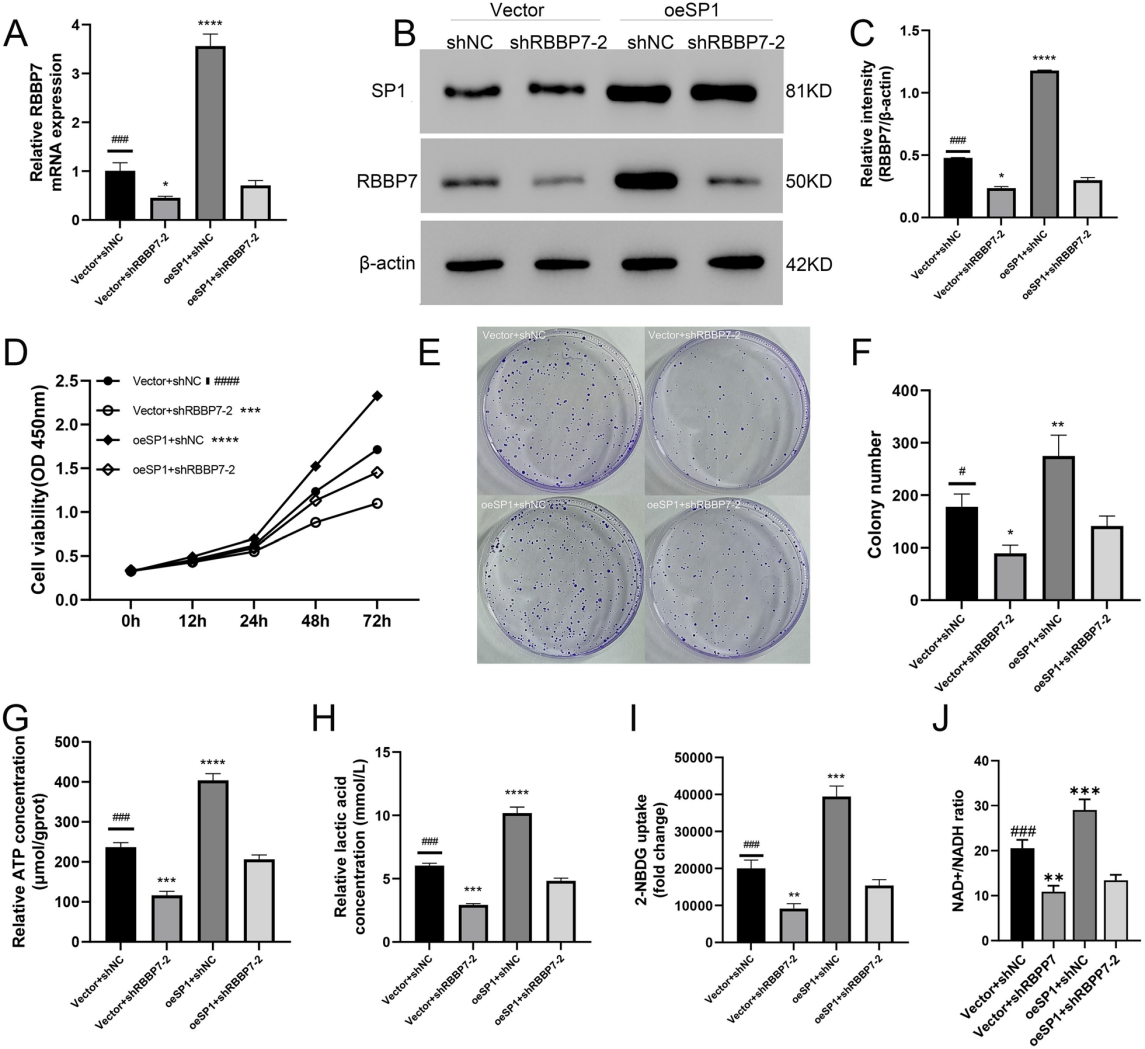
Overexpression of SP1 promotes RBBP7 proliferation and glycolysis in HCC in vitro*.* Compared with oeSP1 + shNC group #, *P* < 0.05; ##, *P* < 0.01; ###, *P* < 0.001; ####, *P* < 0.0001. Compared with oeSP1 + shRBBP7-2 group *, *P* < 0.05; **, *P* < 0.01; ***, *P* < 0.001; ****, *P* < 0.0001. **A**–**C**. RBBP7 expression was test by western blotting and RT-qPCR in SP1 rescue experiments. **D**. CCK-8 assay was employed to examine cell growth in the SP1 rescue experiment. **E**, **F**. Colony formation assay was performed in the SP1 rescue experiment. **G**. ATP concentration in the SP1 rescue experiment. **H**. Lactic acid concentration in the SP1 rescue experiment. **I**. Glucose uptake capacity in the SP1 rescue experiment. **J**. NAD^+^/NADH colorimetric assay in the SP1 rescue experiment

In the oeSP1 + shNC group, both mRNA and protein levels were notably elevated compared to the vector + shNC. (*P* < 0.001) Meanwhile, the Vector + shRBBP7-2 was downregulation when compared to the oeSP1 + shRBBP7-2, (*P* < 0.0001) and oeSP1 + shNC was up-regulated. (*P* < 0.05, Fig. 7A–C) These found imply that oeSP1 would influence RBBP7 mRNA and protein levels.

CCK8 and Colony formation assays demonstrated the promote effect of SP1 in Vector + shNC vs oeSP1 + shNC. And shRBBP7 group reversed the effect, when compared with oeSP1 + shRBBP7-2 group of proliferation, the Vector + shRBBP7-2 group was suppression and the oeSP1 + shNC was promotion (*P* < 0.001, Fig. 7D–F).

SP1 overexpression promoted the cellular ATP levels, lactic acid production, and glucose uptake capacity. Compared to the oeSP1 + shRBBP7 group, the Vector + oeRBBP7 group showed a decrease in the ATP levels, lactic acid production, and glucose uptake capacity, whereas the oeSP1 + shNC group showed an increased in the same (*P* < 0.001, Fig. 7G–J).

### SP1 promotes the effect of *RBBP7* in HCC in vivo

**Fig. 8:**
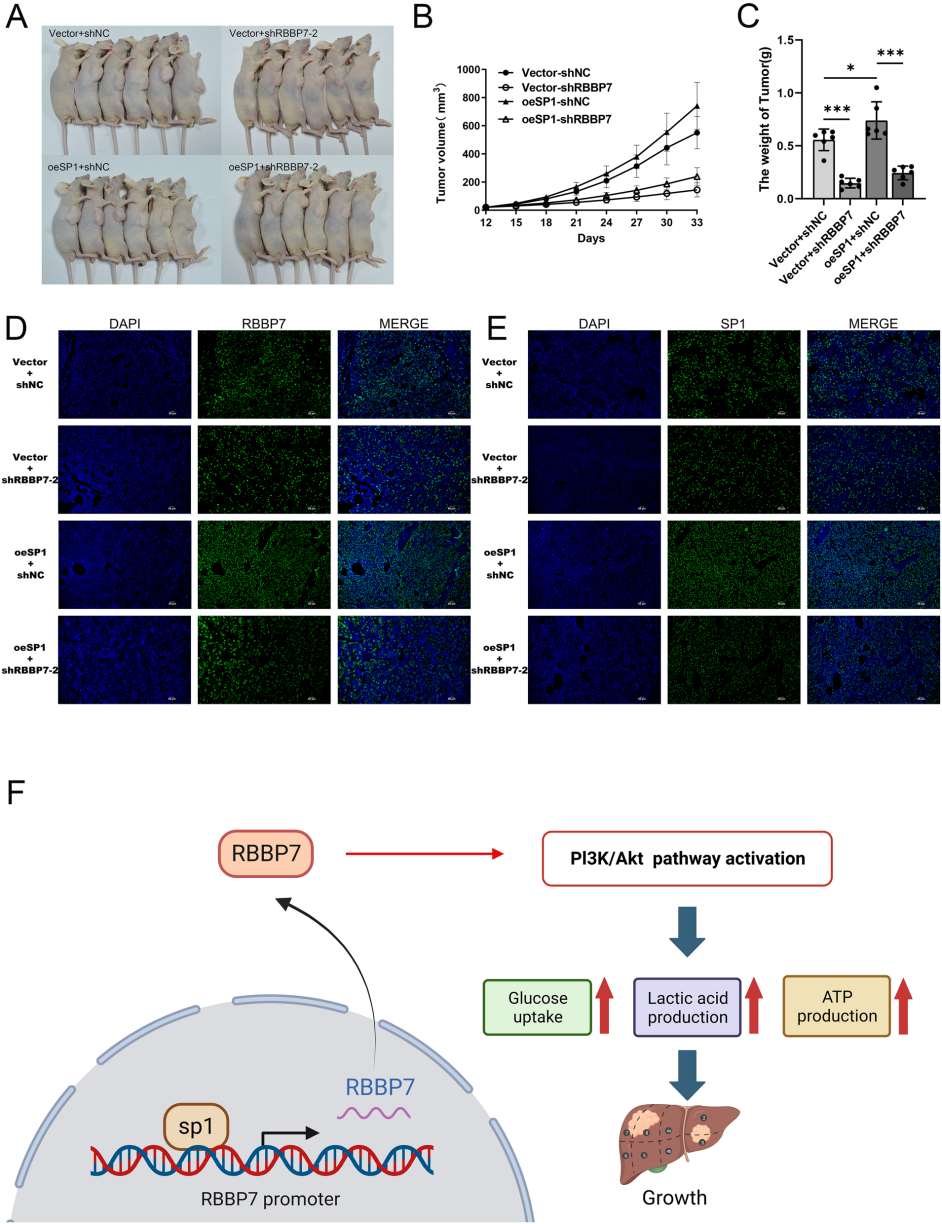
Overexpression of SP1 promotes RBBP7 proliferation and glycolysis in HCC in vivo. **A**. Tumors formed in nude mice injected with Vector + shNC, Vector + shRBBP7-2, oeSP1 + shNC, and oeSP1 + shRBBP7-2 cells. (n = 6). **B**. Volume of tumors formed in different groups. **C**. The weight of the final tumors formed in the various groups. The expression of RBBP7 (**D**), SP1 (**E**), LDHA (Supplemental Fig.2), PFKM (Supplemental Fig.3), and PKM2 (Supplemental Fig.4) in the different groups was measured by immunofluorescence staining. (100x). F. Schematic representation of RBBP7 function and mechanisms of action in HCC

Next, we verified our in vivo findings by injecting shRBBP7 into Hep3B cells with or without SP1 overexpression in nude mice (n = 6) (Fig. 8A). After 33 days, we observed that RBBP7 downregulation could restrict tumor volume and weight compared to the Vector + shNC (*P* < 0.0001). The overexpression of SP1 could promote tumor growth compared to the Vector + shNC. (*P* < 0.05) In the rescue experiment, volume and weight of oeSP1 + shRBBP7-2 group was significantly lower than oesSP1 + shNC. (*P* < 0.0001) (Fig. 8B, C) Additionally, immunofluorescence assay revealed that shRBBP7 could suppress the levels of RBBP7, SP1, PFKM, PKM2, and LDHA in vivo compared with that in the Vector + shNC group. SP1 overexpression could promote the level of PFKM, PKM2, and LDHA compared to the Vector + shNC group. The PFKM, PKM2, and LDHA levels were lower in oeSP1 + shRBBP7 than the oeSP1 + shNC. (Fig. 8D, E, Additional file 1: Figs. S2–S4).

## Discussion

The dysregulation of glucose metabolism is reported to promote the tumorigenesis and progression of HCC [8]. Thus, the elucidation of the molecular mechanism underlying the correlation between glycolysis and tumor cell proliferation may aid in developing strategies to delay tumor progression [16]. This study examined the role of RBBP7 in HCC progression by evaluating its regulatory effects on glycolysis and tumor cell proliferation.

This study demonstrated that RBBP7 is upregulated in HCC using bioinformatics analysis and cellular experiments. These findings were consistent with those of Chen et al. [13] Meanwhile, the area under the ROC curve of RBBP7 was 0.941, indicating that RBBP7 can serve as a diagnostic biomarker for HCC. Furthermore, RBBP7 promoted cell proliferation and glycolysis in HCC. *RBBP7* knockdown downregulated the levels of ATP, lactic acid production, and glucose uptake in HCC, altering the favorable conditions for tumor cell proliferation. Additionally, RBBP7 knockdown markedly downregulated the expression levels of glycolysis-related genes (*PFKM*, *PKM2*, and *LDHA*) [17, 18]. Patients with HCC exhibiting upregulated PKM and PFKM expression levels were associated with increased cumulative recurrence rates when compared with those exhibiting downregulated PKM expression levels [17, 19]. To the best of our knowledge, the role of RBBP7 in aerobic glycolysis has not been previously reported. This is the first study to demonstrate that RBBP7 promotes aerobic glycolysis.

The Warburg effect is a key feature of tumor metabolism during tumor development and progression. Thus, this study examined the signaling pathways regulated by RBBP7 using GSEA. RBBP7 mediated the Warburg effect through the PI3K/AKT signaling, which is a crucial pathway for tumor metabolism [19]. Our results reveal that p-PI3K and p-AKT levels in Hep3B increase in response to RBBP7 overexpression, with the contrasting effect observed upon RBBP7 knockdown in PLCPRF5. T Thus, we hypothesized that RBBP7 enhances the glucose metabolism rate by activating the PI3K/AKT pathway and upregulating PFKM, PKM2, and LDHA expression levels.

Several studies have confirmed that the dysregulation of SP1 contributes to tumor angiogenesis and HCC progression [20, 21]. Using bioinformatics analysis, we predicted that SP1 upregulates RBBP7. We also constructed luciferase reporter vectors containing the presumed complete promoter region of RBBP7. Luciferase assays showed that the transcription factor SP1 is necessary for RBBP7 activation. Results of the ChIP experiment confirmed this observation. Collectively, SP1 facilitated the proliferation of tumor cells and glycolysis in the cells by activating RBBP7, thereby influencing PI3K/AKT signaling.

This study demonstrated that SP1-regulated RBBP7 promotes HCC progression. Additionally, RBBP7 promoted glycolysis and consequently enhanced HCC cell proliferation. For translational value, the findings of this study suggest that RBBP7 is a potential diagnostic biomarker for HCC.

This study demonstrated that the SP1/RBBP7/PI3K/AKT axis promotes HCC progression by activating glycolysis. The presence of other regulatory mechanisms involved in upregulating RBBP7 expression in HCC was not determined, which is a limitation of this study and must be validated in future studies. Further studies must be performed with increased numbers of clinicopathological samples, incorporating RBBP7 expression into the prediction model. After multi-center verification, the prediction model can be applied in the clinics on a large scale.

## Conclusion

Our previous investigation showed that RBBP7 is expressed at high levels in HCC, and that its upregulation is significantly related to the proliferation and glycolysis in HCC. This phenomenon aligns with the Warburg effect, a well-known hallmark of tumor metabolism that contributes to tumorigenesis and the progression of tumors. We explored that SP1 could activate RBBP7 and influence PI3K/AKT signaling. The findings of this study will aid in the development of novel diagnostic and therapeutic strategies for HCC.

### Supplementary Information

**Additional file 1: Fig S1.** Correlation analysis with RBBP7. **Fig S2.** The expression of LDHA in the different groups was measured by immunofluorescence staining. (100x). **Fig S3.** The expression of PFKM in the different groups was measured by immunofluorescence staining. (100x). **Fig S4.** The expression of PKM2 in the different groups was measured by immunofluorescence staining. (100x). **Table S1.** Antibody. **Table S2.** ShRNA-RBBP7 sequence

## Data Availability

The data used to support the findings of this study are available from the corresponding author upon request.
