# Supplementary material for: RBBP7, regulated by SP1, enhances the Warburg effect to facilitate the proliferation of hepatocellular carcinoma cells via PI3K/AKT signaling
Source: J Transl Med. 2024 Feb 18;22:170. doi: 10.1186/s12967-024-04964-8 (PMC10874528; doi:10.1186/s12967-024-04964-8)
Supplement: Supplementary file 1 — Additional file 1: Fig S1. Correlation analysis with RBBP7. Fig S2. The expression of LDHA in the different groups was measured by immunofluorescence staining. (100x). Fig S3. The expression of PFKM in the different groups was measured by immunofluorescence staining. (100x). Fig S4. The expression of PKM2 in the different groups was measured by immunofluorescence staining. (100x). Table S1. Antibody. Table S2. ShRNA-RBBP7 sequence [file 12967_2024_4964_MOESM1_ESM.docx]

Additional file 1: Fig. S1 Correlation analysis with RBBP7.


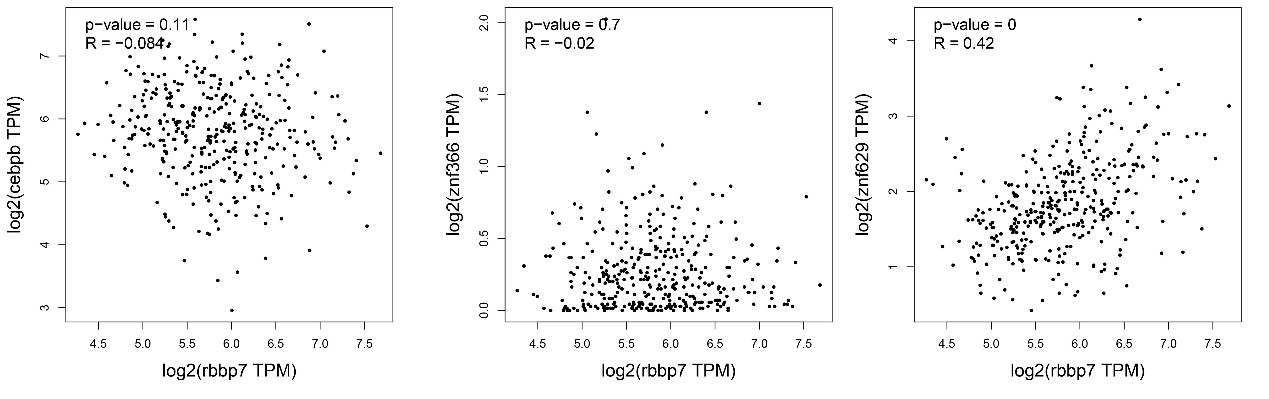


Additional file 1: Fig. S2 The expression of LDHA in the different groups was measured by immunofluorescence staining. (100x).


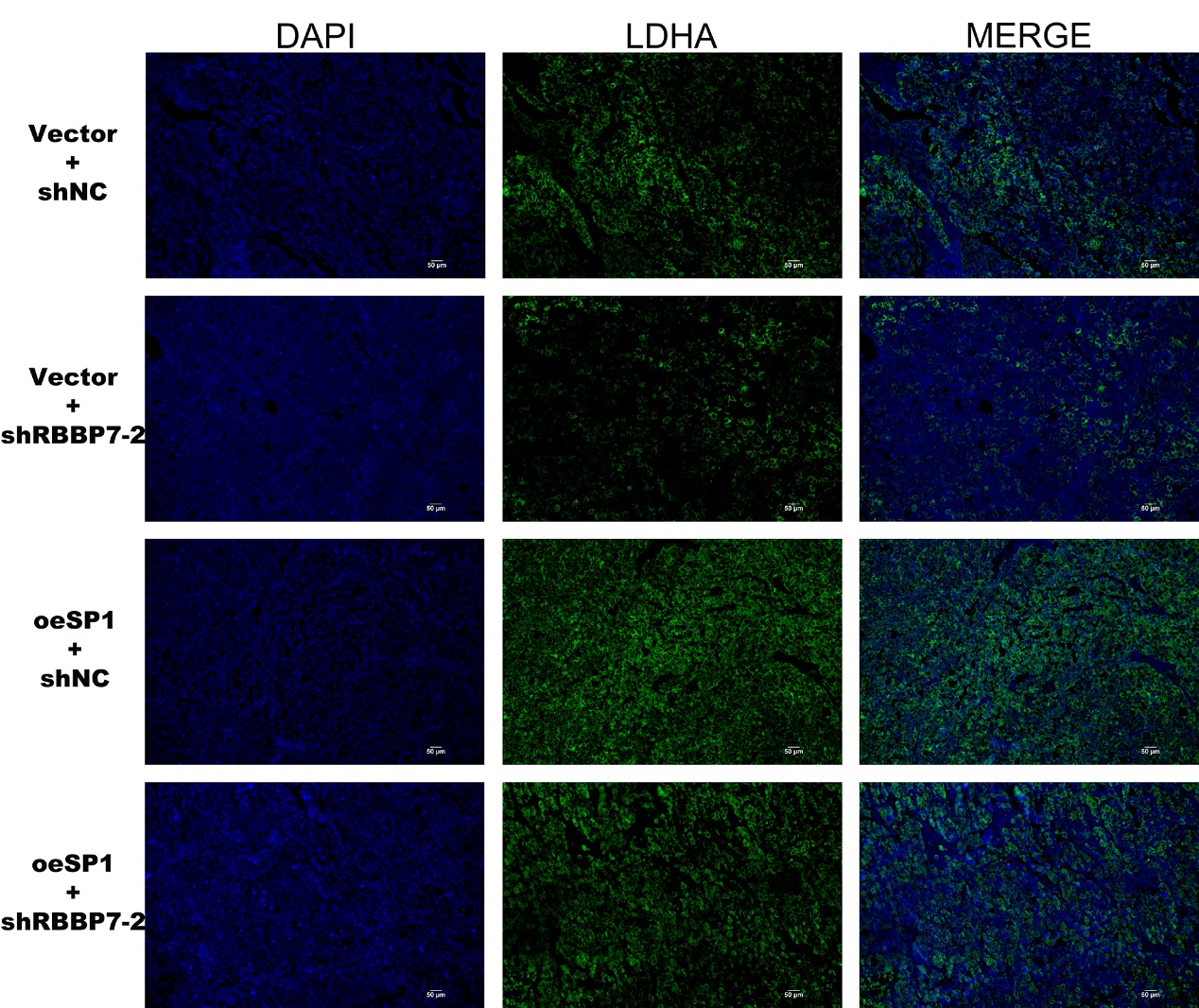


Additional file 1: Fig. S3 The expression of PFKM in the different groups was measured by immunofluorescence staining. (100x).


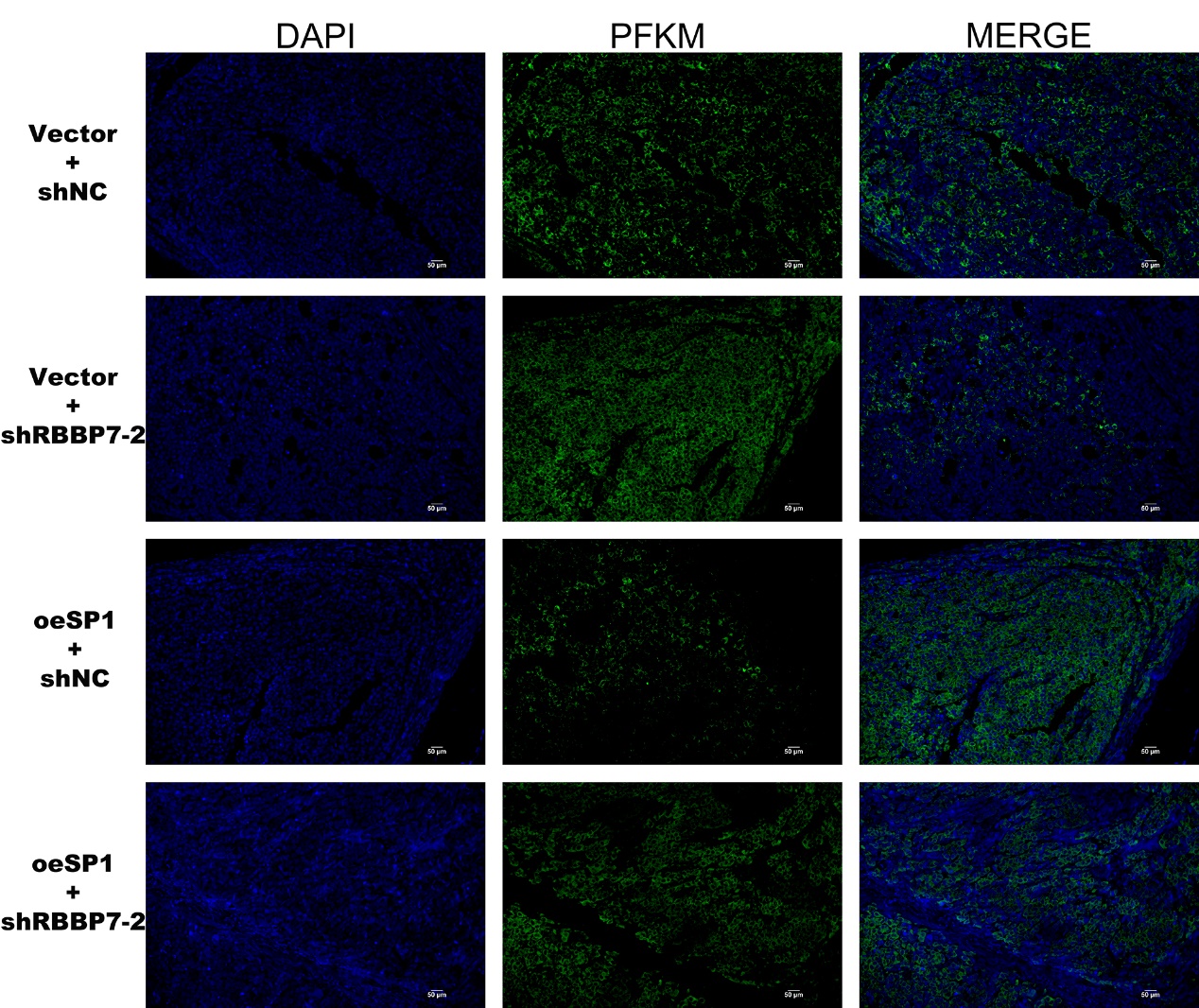


Additional file 1: Fig. S4 The expression of PKM2 in the different groups was measured by immunofluorescence staining. (100x).


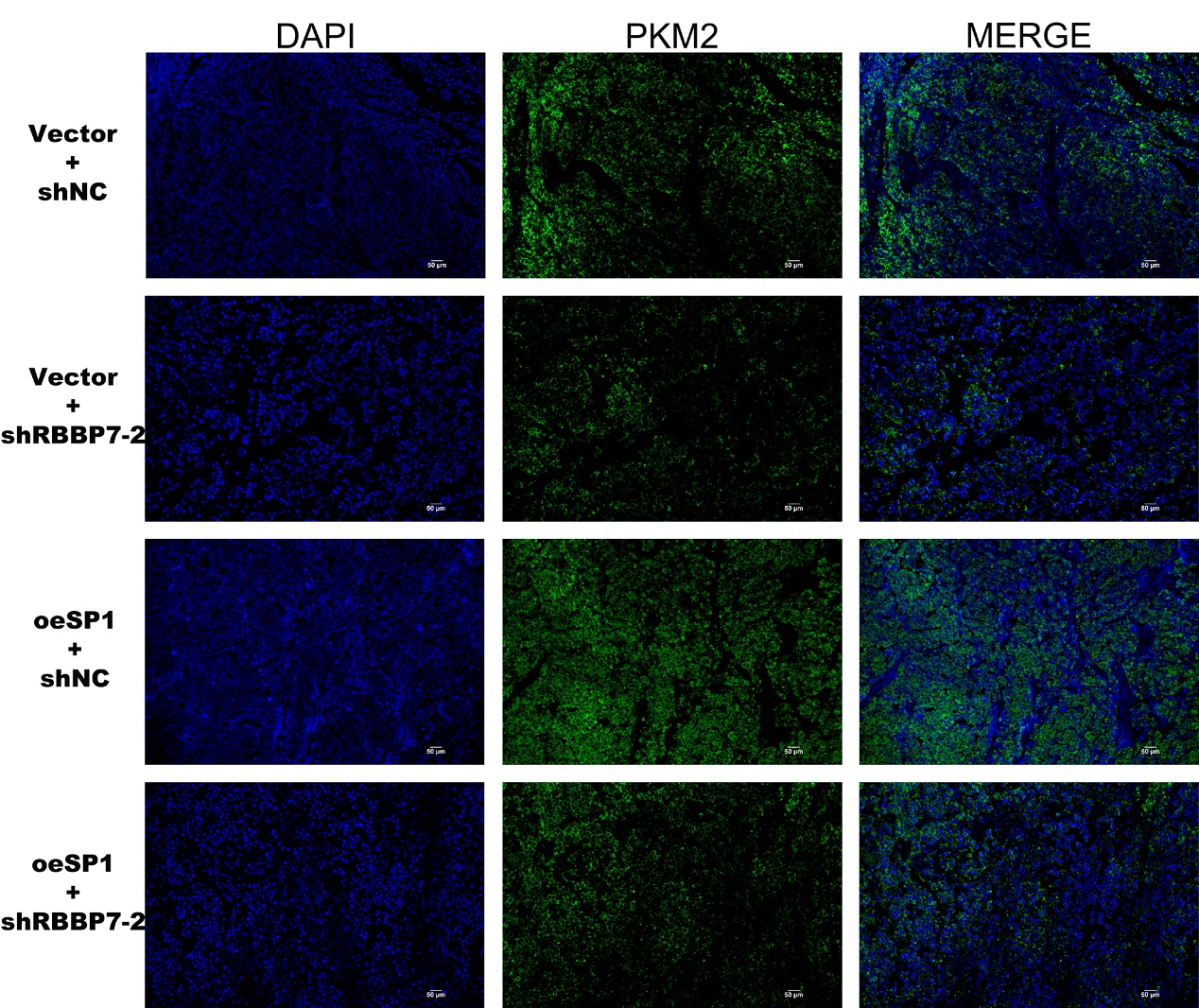


Additional file 1: Table S1 Antibody

| Gene Name | Company | Catalog Number | Dilution |
| --- | --- | --- | --- |
| RBBP7 | Abcam | Ab259957 | 1:1000 |
| p-PI3K | CST | 17366 | 1:1000 |
| PI3K | CST | 4292 | 1:1000 |
| p-AKT | CST | 4060 | 1:1000 |
| AKT | CST | 9272 | 1:1000 |
| PFKM | Abcam | Ab154804 | 1:1000 |
| PKM2 | Abcam | Ab85555 | 1:800 |
| LDHA | Abcam | Ab52488 | 1:5000 |
| SP1 | Abcam | Ab157123 | 1:5000 |
| β-actin | Proteintech | 66009-1-Ig | 1:5000 |
| HRP-conjugated Affinipure Goat Anti-Rabbit IgG | Proteintech | SA00001-2 | 1:10000 |
| HRP-conjugated Affinipure Goat Anti-Mouse IgG | Proteintech | SA00001-1 | 1:10000 |

Additional file 1: Table S2 ShRNA-RBBP7 sequence

| Characteristic |  |
| --- | --- |
| shRBBP7-1 | SS Seq: 5’-GGATAAGACCGTAGCTTTA-3’  AS Seq: 5’-TAAAGCTACGGTCTTATCC-3’ |
| shRBBP7-2 | SS Seq: 5’-GCTGATGATCAGAAACTTA-3’  AS Seq: 5’-TAAGTTTCTGATCATCAGC-3’ |
| shRBBP7-3 | SS Seq: 5’-GGAAGAACAATCAGCAGAA-3’  AS Seq: 5’-TTCTGCTGATTGTTCTTCC-3’ |
